# Rumen Microbiome and Metabolome of High and Low Residual Feed Intake Angus Heifers

**DOI:** 10.3389/fvets.2022.812861

**Published:** 2022-03-25

**Authors:** Yue Liu, Hao Wu, Wanbao Chen, Chang Liu, Qingxiang Meng, Zhenming Zhou

**Affiliations:** State Key Laboratory of Animal Nutrition, College of Animal Science and Technology, China Agricultural University, Beijing, China

**Keywords:** rumen, microbiota, metabolomics, residual feed intake, beef cattle

## Abstract

Feed cost is the greatest expense during cattle production; therefore, reducing it is critical to increasing producer profits. In ruminants, the microbial population is important to nutrient digestion and absorption in the rumen. The objective of this study was to investigate the relationships among rumen bacteria, rumen metabolites, and the residual feed intake (RFI) phenotype of beef cattle. Twelve Angus heifers were selected to be sampled and divided into high RFI (HRFI; *n* = 6) group and low RFI (LRFI; *n* = 6) group according to their RFI classification determined during the feedlot-finishing period. After the ruminal liquid samples were collected at slaughter, Illumina MiSeq sequencing of the 16S rRNA V3-V4 region and liquid chromatography-mass spectrometry (LC-MS) were performed to determine their bacterial composition and metabolites, respectively. At the phylum level, the relative abundance of Proteobacteria was higher in the LRFI group than in the HRFI group (*P* < 0.01). At the family level, the relative abundances of *Rikenellaceae* (*P* < 0.01), *Ruminococcaceae, Bacteroidales_S24-7_group*, and *Lachnospiraceae* (*P* < 0.05) were significantly higher in the LRFI group. At the genus level, the relative abundances of *Rikenellaceae_RC9_gut_group* and *Ruminiclostridium_1* were higher in the LRFI group (*P* < 0.01), as were the relative abundances of *norank_f__Bacteroidales_S24-7_group, Lachnospiraceae_ND3007_group*, and *Lachnospiraceae_NK3A20_group* (*P* < 0.05). Moreover, the genera *Rikenellaceae_RC9_gut_group, Ruminococcaceae_NK4A214_group, Christensenellaceae_R-7_group, Ruminococcaceae_UCG-010, Lachnospiraceae_ND3007_group, Ruminiclostridium_1*, and *Lachnospiraceae_NK3A20_group* were negatively associated with the RFI; both foundational and key species are associated with feed efficiency phenotype. In addition, rumen metabolomics analysis revealed that the RFI was associated with significantly altered concentrations of rumen metabolites involved in protein digestion and absorption, Linoleic acid metabolism, Lysine degradation, and Fatty acid degradation. Correlation analysis revealed the potential relationships between the significantly differential ruminal metabolites and the genera ruminal bacteria. The present study provides a better understanding of rumen bacteria and metabolites of beef cattle with different RFI phenotypes and the relationships among them, which are potentially important for the improvement of beef cattle feed efficiency.

## Introduction

In beef cattle production systems, feed costs represent about 60–75% of the total cost (1), requiring beef cattle producers to pay attention to the feed efficiency trait (2). The traditional expression method is the relationship between beef cattle weight gain and feed intake, such as the Gain-to-Feed Ratio (G/F) and the Feed-to-Gain Ratio (F/G). These are the ratio of two traits; therefore, there are some problems in their practical application. For example, the G/F will have the same genetic background but with low consumption and low output, and high consumption and high output, and these two types of individuals will have huge differences. While the F/G, individuals with lower feed conversion rate (FCR) grow faster because they maintain their basal metabolism and energy needs by increasing their food intake (3). In view of this, the concept of residual feed intake (RFI) was proposed by Koch et al. (4). RFI is defined as the difference between the actual dry matter intake (DMI) and the predicted DMI based on body size and growth (1, 5). RFI correlates significantly with the FCR and DMI, but not with the average daily gain (ADG) (6), indicating that the RFI is independent of animal body weight gain and growth rate. Moreover, RFI is a negative selection trait (7), the lower RFI, the higher the feed efficiency. Animals with a low RFI (LRFI) typically consume less feed than animals with a high RFI (HRFI) (6), resulting in maximized profitability of the beef industry (1). Some studies have demonstrated that LRFI animals not only have greater diet digestibility, but also lower methane emissions (8), thus selection for LRFI might also be a great strategy for greenhouse gas mitigation. Additionally, the RFI has moderate heritability: 0.18–0.43 (7), suggesting that it is also influenced by non-genetic factors (9).

Rumen microbial fermentation produces volatile fatty acids (VFAs) and microbial proteins (10), among which VFAs provide ~70% of the energy required by the host (11). Nkrumah et al. (5) observed that the energy metabolism of beef cattle with different RFIs was significantly different. Herd and Arthur (8) reported that ruminant digestion and rumen fermentation can explain 19% of RFI changes. These results indicated that the rumen microbial population composition might be related to the RFI phenotypes. Later, studies showed that there is indeed a correlation between the rumen microbiome and the RFI phenotype in dairy cows (12–14) and beef cattle (15). However, the correlation between bacteria and RFI phenotype is controversial. Furthermore, Paz et al. (16) reported that the rumen microbiome could explain about 20% of the changes in feed efficiency traits of beef steers. Although links between the rumen microbiome and host feed efficiency have been identified, the mechanisms driving these changes are unclear, and it is unknown whether foundational or keystone species are responsible for the phenotypic differences in feed efficiency. Additionally, rumen microbes produce metabolites, which are released into the rumen lumen and are absorbed through the rumen epithelium or the epithelium in the small intestinal tract (17). For these metabolites, differences in their production, as well as variation in their absorption, might result in variation in nutrient utilization and efficiency of ruminants, ultimately leading to physiological or phenotypic changes (18, 19). To date, for RFI phenotypes in beef cattle, experiments aiming to determine the relationships among the RFI phenotype, rumen microbiota, and metabolites are yet to be undertaken.

The aim of this study was to identify the relationships among the RFI phenotype of Angus heifers, rumen microbiota, and rumen metabolites. We hypothesized that beef cattle with different RFI values would have distinct rumen bacteria and metabolites, and potential relationships might be between rumen bacteria and metabolites.

## Materials and Methods

### Animals, Diet, and Calculation of RFI

The Angus heifers used in this study were maintained according to the guidelines of the Laboratory Animal Welfare and Animal Experiment Ethical Committee of China Agricultural University (Protocol No. AW08059102-2). Forty-two Angus heifers (410 ± 25 kg body weight, aged 15 months) were fed with a diet containing 50% concentrate and 50% forage (Supplementary Table 1) for 144 days (21 days of adaptation to feedlot diet and the environment, followed by 123 days of data collection) according to NRC (20). During the experiment, all conditions were consistent. Feeding tank automatic identification of each animal's electronic ear tag, which records feed intake per time (Zhenghong Agriculture and Animal Husbandry Machinery and Equipment Co, Shanghai, China) was used to obtain the daily feed intake of each animal. All heifers had *ad libitum* access to water and feed during the experimental period. Weight measurements of all heifers were performed at the beginning and the end of the experiment as well as at 14-d intervals for 123 d. Each heifer's average daily gain (ADG) during the experiment was computed as the coefficient of the linear regression of body weight (BW; kg) on time (d) using the PROC REG component of the SAS package (SAS Inst., Inc., Cary, NC, USA). The metabolic body weight (MBW) of each heifer over the experimental period was computed as the midtest BW^0.75^ of the 123-d test (21). The total actual DMI of each heifer was divided by the test period of 123 d to give an average actual DMI. The expected DMI of each heifer over the test period was modeled and predicted by using the MBW, ADG and actual DMI with PROC REG (21). The residual feed intake (RFI) was defined as the difference between the actual and expected DMI using the following model (22):


$$\begin{array}{l} \text{DMI }=\;{\text{β}}_{0}\;+\;{\text{β}}_{1}\text{MBW }+\;{\text{β}}_{2}\text{ ADG }+\text{ ε} \end{array}$$

in which β_0_ is the y-intercept, β_1_ is the regression coefficient of MBW, β_2_ is the regression coefficient of ADG, and ε is the RFI. RFI Standard deviations above and below the mean were used to group animals into high (> 0.5 SD) and low RFI (<0.5 SD) groups (21).

### Heifer Selection, Collection of Ruminal Liquid Samples, and Determination of Fermentation Parameters

Heifers were ordered based on their RFI values, and the six least efficient (HRFI) and the six most efficient (LRFI) heifers were selected. Specifically, the RFI values and animal performance can be found in Table 1, Supplementary Table 2, and Supplementary Table 3. Heifers had *ad libitum* access to water but were fasted prior to slaughter the following morning. After slaughter, the ruminal liquid samples were collected from the ruminal ventral sac. The pH was measured using a portable pH meter (PHS-3C, Shanghai Leici Instrument Factory, Shanghai, China) immediately. One hundred milliliters of the samples were centrifuged at 10,000 × *g* for 10 min at 4°C to obtain the supernatant, which was used to determine the concentration of NH_3_-N and VFAs using a spectrophotometer (UV-VIS 8500, Tianmei Scientific Instrument Co., Shanghai China) and gas chromatography (SP-3420, Beijing Analytical Instrument Factory, Beijing, China), respectively. The other parts of samples were frozen immediately in liquid nitrogen, and stored at −80°C until subsequent microbial DNA extraction and metabolomic analysis.

**Table 1:** Performance of Angus heifers according to RFI groups.

**Items^a^**	**HRFI**	**LRFI**	**SEM^b^**	***P*-value^c^**
No. animals	6	6	-	-
Initial weight, kg	425.61	412.61	5.41	0.12
DMI, kg/d	9.51	7.66	0.33	<0.01
ADG, kg/d	0.88	1.01	0.08	0.28
RFI, kg/d	0.95	−1.33	0.18	<0.01

a *HRFI, high residual feed intake; LRFI, low residual feed intake; DMI, dry matter intake; ADG, average daily gain*.

b *SEM, standard error of the mean*.

c *P-values were derived using a Student's t-test to assess the differences between the HRFI and LRFI groups*.

### DNA Extraction, 16S rRNA Gene Amplification, and Sequencing

Microbial DNA of 12 ruminal liquid samples was extracted from the rumen samples using an E.Z.N.A.® soil DNA Kit (Omega Bio-tek, Norcross, GA, USA) following the manufacturer's protocols. The V3–V4 hypervariable regions of the bacterial 16S rRNA gene were amplified using the primer pairs 338F (5′- ACTCCTACGGGAGGCAGCAG-3′) and 806R (5′-GGACTACHVGGGTWTCTAAT-3′) in a thermocycler PCR system (GeneAmp 9700, ABI, Foster City, CA, USA). All PCR reactions were performed in triplicate in a total reaction volume of 20 μl, containing 4 μl of 5 × FastPfu Buffer, 10 ng of DNA, 2 μl of 2.5 mM dNTPs, 0.8 μl of each Forward Primer (5 μM) and Reverse Primer (5 μM), 0.4 μl of FastPfu Polymerase, and 0.2 μl of bovine serum albumin (BSA). After electrophoresis, the amplified products were extracted from 2% agarose gels, purified using an AxyPrep DNA Gel Extraction Kit (Axygen Biosciences, Union City, CA, USA), and quantified using QuantiFluor™-ST (Promega, Madison, WI, USA) following the manufacturer's protocols. Paired-end sequencing libraries (2 ×300 bp) were constructed by Majorbio Bio-Pharm Technology Co. Ltd. (Shanghai, China). Purified amplicons were pooled in equimolar amounts and all libraries were sequenced on an Illumina MiSeq platform (Illumina, San Diego, CA, USA) at Majorbio Bio-Pharm Technology Co. Ltd. according to standard protocols (23).

### Sequence Processing and Analysis

The raw sequences obtained from the MiSeq platform were quality-filtered using fastp version 0.20.0 (24) and merged using FLASH version 1. 2. 7 (25) with the following criteria: (1) The reads were truncated at any site receiving an average quality score of <20 over a 50-bp sliding window; (2) sequences were merged with overlaps longer than 10 bp (maximum error ratio = 0.2); (3) sequences of each sample were separated according to barcodes (exactly matching) and primers (allowing two nucleotide mismatches), and reads containing ambiguous characters were discarded. Sequences were binned into operational taxonomic units (OTUs) at 97% similarity using UPARSE version 7.1 with a confidence threshold of 0.70, and the taxonomy of each OTU representative sequence was analyzed using the RDP Classifier version 2.2 against the Silva 128/16S_bacteria database (26, 27). Chimera-filtering and OTU-clustering were performed simultaneously using a novel “greedy” algorithm (28, 29). Analyses was performed using the Majorbio I-Sanger Cloud Platform (www.i-sanger.com). Alpha diversity indexes were assessed using MOTHUR version v.1.30.1 (30). The bar graphs were analyzed using the “vegan” package in the R software (31). Beta-diversity was estimated by computing the Bray_Curtis distance, calculated as similarities (ANOSIM) (999 permutations), and visualized using principal coordinate analysis (PCoA) by the “vegan” package in R (31). Significant differences in the abundance of the microbiota at the phylum, family, and genera levels between the high residual feed intake (HRFI) and low residual feed intake (LRFI) groups were identified using Student's *t*-test and by false discovery rate (FDR) multiple check calibration by the stats package in R, together with the scipy package in python (32–34).

### LC-MS Metabolomic Processing

All rumen samples were analyzed using the LC-MS platform (Thermo Ultimate 3000LC, Q Exactive; ThermoFisher Scientific, Waltham, MA, USA). Briefly, 100 μl of each sample was weighed accurately, then 400 μl of methanol/water (4:1 v/v) was used to extract the metabolites. The mixture was allowed to settle at −20°C, treated using a high throughput tissue grinder (Wonbio-96, Shanghai Wanbo biotechnology Co., Ltd., Shanghai, China) at 50 Hz for 6 min, vortexed for 30 s, and then with ultrasound at 40 kHz for 30 min at 5°C. The samples were incubated at −20°C for 30 min. After centrifugation at 13,000 × *g* for 15 min at 4°C, the supernatant was used for LC-MS/MS analysis. Chromatographic separation of the metabolites was performed on the ExionLC^TM^AD system (AB Sciex, Framingham, MA, USA) equipped with an ACQUITY UPLC HSS T3 column (100 ×2.1 mm i.d., 1.8 μm particle size; Waters, Milford, MA, USA). The mobile phases consisted of solvent A: 0.1% formic acid in water with formic acid (0.1%) and solvent B: acetonitrile 50% and isopropyl alcohol 50% with 0.1% formic acid. The solvent gradient of the mobile phase (A:B) consisted of the following: from 0 to 3 min, 95%:5% to 80%:20%; from 3 to 9 min, 80%:20% to 5%:95%; from 9 to 13 min, 5%:95% to 5%:95%; from 13.0 to 13.1 min, 5%:95% to 95%:5%; and from 13.1 to 16.0 min, 95%:5% to 95%:5% to equilibrate the system. The UPLC system was coupled to a quadrupole-time-of-flight mass spectrometer (Triple TOFTM5600+, AB Sciex) equipped with an electrospray ionization (ESI) source. The source temperature was 500°C, the curtain gas (CUR) was 30 psi, on-spray voltage floating (ISVF) was carried out in negative mode (−4000 V) and positive mode (5000 V), the declustering potential was 80 V, and the MS/MS rolling was 20–60 V. To test the repeatability of the system, quality control (QC) samples prepared by mixing equal volumes of all ruminal liquid were injected at regular intervals.

### Metabolomics Data Analysis

Raw data from the UPLC/MS analysis were first imported into the Progenesis QI 2.3 format (Nonlinear Dynamics, Waters) for baseline filtering, peak recognition, integration, retention time correction, and peak alignment. Finally, a data matrix of retention time, mass charge ratio, and peak intensity was obtained. For the missing values, at least 50% of the metabolic features of samples were retained, the vacancy values were filled (the minimum value in the original matrix), and the metabolic features were normalized by the summation normalization method. After discarding the relative standard deviation (RSD) of QC >30%, the final matrix was obtained. Then, statistical analysis was performed on log_10_ transformed data to identify significant differences in metabolite levels between groups. All data were visualized between the HRFI and LRFI groups using principle component analysis (PCA), followed by orthogonal partial least squares discriminant analysis (O)PLS-DA with Student's *t*-test and the following screening criteria: Variable importance in the projection (VIP) values > 1.0, difference multiple [fold change (FC)] > 1.0 or FC <1.0 and *P* <0.05 to obtain significantly differentially abundant metabolites between the LRFI and HRFI groups. Additionally, significantly differentially abundant metabolites were analyzed for abundance pattern clustering using the gplots package in R (35). The impact of the RFI on metabolic pathways and metabolite set enrichment were analyzed using the Stats package in R and the scipy package in python (32, 33). Correlations among the rumen genera bacteria, significantly differentially abundant rumen metabolites, and the RFI phenotype were assessed using Spearman's correlation analysis in the pheatmap package in R (36).

## Results

### Animal Performance

Table 1 shows the growth performance of HRFI and LRFI heifers during the experimental period. At the beginning of the experiment, the HRFI and LRFI heifers were not different in initial weight (*P* = 0.12). However, the LRFI heifers had a lower DMI and lower RFI value compared with the HRFI heifers (*P* <0.01).

### Rumen Fermentative Parameters

The ruminal liquid pH; the concentrations of NH_3_-N and the total VFAs (TVFAs); the proportion of acetate, propionate, butyrate, valerate, isobutyrate, and isovalerate; and the ratio of acetate:propionate (AP) of the Angus heifers did not vary between the HRFI and LRFI groups (*P* > 0.05; Table 2).

**Table 2:** Rumen fermentative parameters of Angus heifers according to RFI groups.

**Items^a^**	**HRFI**	**LRFI**	**SEM^b^**	***P-*value^c^**
pH	6.63	6.86	0.09	0.10
NH_3_-N, mg/dl	10.40	11.07	0.98	0.64
TVFA, mM	58.89	62.09	4.64	0.64
**VFAs, molar % of TVFA**
Acetate	71.35	70.99	0.22	0.33
Propionate	16.04	16.41	0.40	0.54
Butyrate	9.77	9.68	0.19	0.77
Valerate	0.55	0.60	0.02	0.12
Isobutyrate	0.99	0.93	0.13	0.73
Isovalerate	1.30	1.39	0.13	0.64
AP	4.47	4.34	0.11	0.44

a *HRFI, high residual feed intake; LRFI, low residual feed intake; VFAs, volatile fatty acids; TVFAs, total VFAs; AP, the acetate-to-propionate ratio*.

b *SEM, standard error of the mean*.

c *P-values were derived using a Student's t-test to assess the differences between the HRFI and LRFI groups*.

### Sequencing, and Alpha and Beta Diversity Analyses

**Figure 1:**
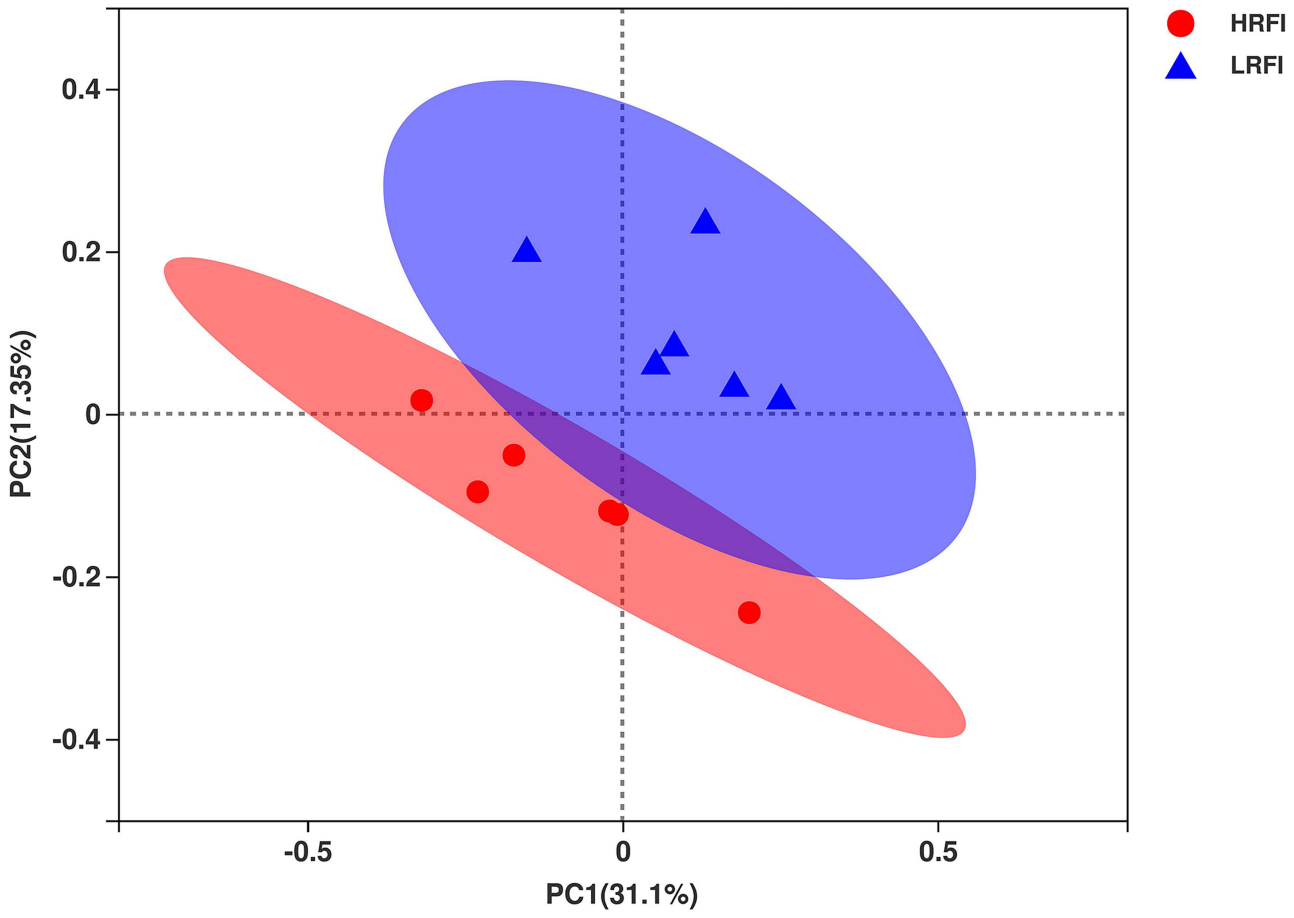
The principal coordinate analysis (PCoA) of the ruminal bacterial communities. Individual points represent a ruminal sample and colors represent RFI groups.

In total, 657,130 raw bacterial sequences were obtained from 12 samples. After quality control to an equal sequencing depth (24,226 reads per sample) and clustering, we obtained 1,908 OTUs at the 97% similarity level, which were assigned to 19 phyla, 37 classes, 62 orders, 94 families, and 214 genera. Good's coverage after normalization for the samples was > 98.8% for the bacterial community, indicating sufficient sequence coverage for the samples. Chao1's richness, Shannon's diversity, and Simpson's diversity of Alpha diversity demonstrated that the bacterial community of Angus heifers did not vary between the RFI groups (*P* > 0.05; Supplementary Table 4). The PCoA plot showed bacterial communities clustered by RFI phenotype, which clearly demonstrated the distinct bacterial community structure in the HRFI and LRFI groups (Figure 1), indicating that the RFI phenotype influences the bacterial community composition.

### Bacterial Abundance

The bacterial community was dominated by the phyla Bacteroidetes (66.71%, 61.65%) and Firmicutes (28.27%, 33.89%) in the HRFI group and LRFI group, respectively (Supplementary Figure 1A). The most abundant families in the HRFI group included the *Prevotellaceae* (36.52%), *Rikenellaceae* (11.55%), and *Ruminococcaceae* (10.67%). By contrast, the *Prevotellaceae* (20.10%), *Rikenellaceae* (18.57%), *Ruminococcaceae* (14.50%) and *Bacteroidales_BS11_gut_group* (12.18%) were present in the greatest abundance in the LRFI group (Supplementary Figure 1B). At the genus level, the predominant genera in the HRFI group were *Prevotella_1* (29.18%) and *Rikenellaceae_RC9_gut_group* (11.00%), while in the LRFI group, the predominant genera were *Rikenellaceae_RC9_gut_group* (17.62%), *Prevotella_1* (14.48%), and *norank_f_Bacteroidales_BS11_gut_group* (12.18%) (Supplementary Figure 1C).

### Significantly Differentially Abundant Rumen Bacteria

**Figure 2:**
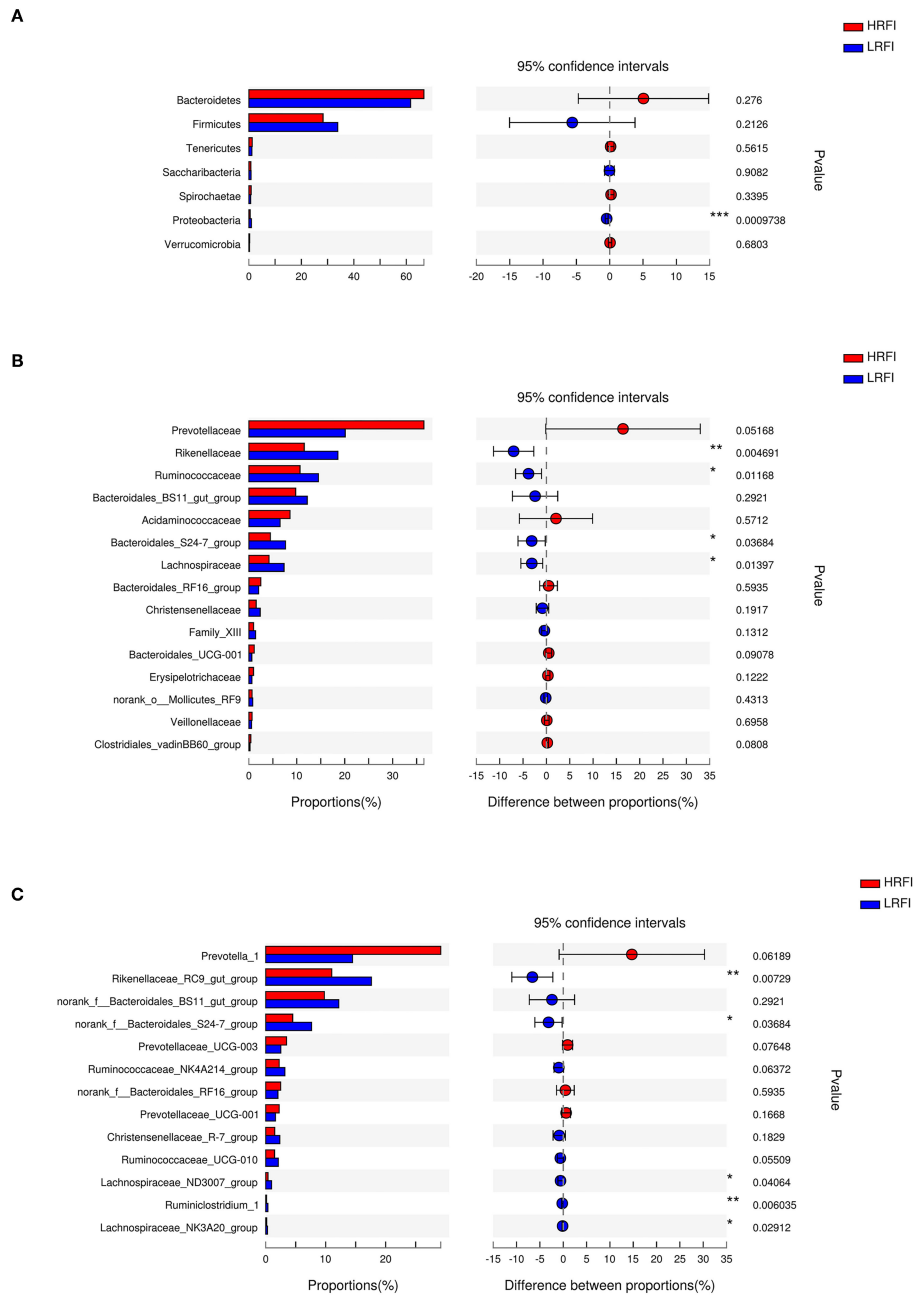
Classification of the ruminal bacterial community composition across the HRFI and LRFI groups. Extended error bar plot showing the significantly different phyla **(A)**, families **(B)**, and genera **(C)** (relative abundance > 0.1% for all samples). Positive and negative differences indicate a greater abundance in the HRFI group and LRFI group, respectively. Asterisks indicate significant difference between the HRFI and LRFI groups (***P ≤ 0.001, **0.001 < *P* ≤ 0.01; *0.01 < *P* ≤ 0.05).

At the phylum level (Figure 2A), the relative abundance of Proteobacteria (0.48%, 0.94%) was higher in the LRFI group (*P* <0.01). At the family level (Figure 2B), the relative abundance of *Rikenellaceae* (11.55%, 18.57%; *P* <0.01), *Ruminococcaceae* (10.67%, 14.50%; *P* <0.05), *Bacteroidales_S24-7_group* (4.49%, 7.64%; *P* <0.05), *Lachnospiraceae* (4.22%, 7.34%; *P* <0.05) were higher in the LRFI group, while the relative abundances of *Prevotellaceae* (36.52%, 20.10%) and *Bacteroidales_UCG-001* (1.12%, 0.61%) tended to be higher in the HRFI group (*P* <0.10). At the genus level (Figure 2C), the relative abundances of *Rikenellaceae_RC9_gut_group* (11.01%, 17.62%; *P* <0.01), *Ruminiclostridium_1* (0.15%, 0.36%; *P* <0.01), *norank_f__Bacteroidales_S24-7_group* (4.49%, 7.64%; *P* <0.05), *Lachnospiraceae_ND3007_group* (0.39%, 0.97%; *P* <0.05), and *Lachnospiraceae_NK3A20_group* (0.15%, 0.29%; *P* <0.05) were higher in the LRFI group. Moreover, the relative abundances of *Ruminococcaceae_NK4A214_group* (2.24%, 3.19%), and *Ruminococcaceae_UCG-010* (1.47%, 2.09%) tended to be higher in the LRFI group (*P* <0.10), while the relative abundances of *Prevotella_1* (29.18%, 14.48%), and *Prevotellaceae_UCG-003* (3.47%, 2.53%) tended to be higher in the HRFI group (*P* <0.10).

### Rumen Metabolomic Profiling

#### Sample Quality Control

**Figure 3:**
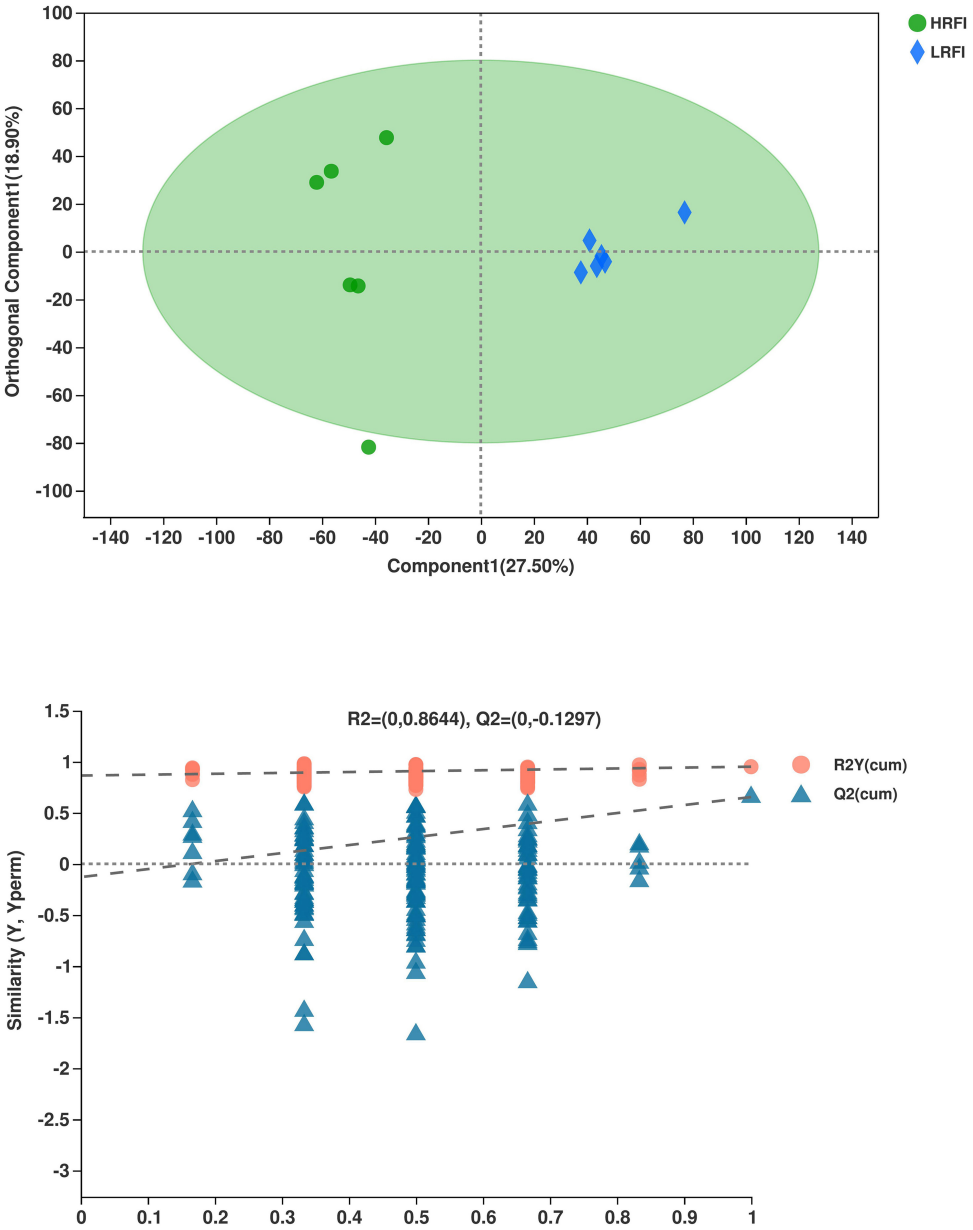
The orthogonal partial least squares discriminant analysis (OPLS-DA) plot of rumen metabolites in comparisons of the HRFI and LRFI groups.

The overlap of the total ion chromatogram of the QC sample in the positive (A) and negative (B) ion modes are shown in Supplementary Figure 2. The results confirmed the stability and reproducibility of the data obtained in this study. PCA provided a satisfactory separation of the data between the two groups (Supplementary Figures 3A,B, respectively). Validation plots in Figure 3 show the parameters for the assessment of the OPLS-DA model quality in discriminating the HRFI and LRFI groups. All the samples in the plots were within the 95% Hotelling T2 ellipse, while only one sample in the HRFI group was outside the ellipse. The permutation test of the group was in a better range, with the *R*^2^-value of 0.864 indicating moderate effectiveness of the model.

#### Significantly Differentially Abundant Rumen Metabolites

As shown in Table 3, there were 11 common differential metabolites between the LRFI and HRFI groups, which were identified using a VIP threshold of one (*P* <0.05). Among the 11 metabolites, four were classified as associated with protein digestion and absorption, three with linoleic acid metabolism, three with Lysine degradation, and one with fatty acid degradation.

**Table 3:** Significantly differentially ruminal metabolites of Angus heifers in the comparison between the LRFI and HRFI groups^a^.

**No**.	**Metabolites**	**Formula**	**VIP^b^**	***P*-value^c^**	**Fold change**	**Trend^d^**
**Protein digestion and absorption**						
1	L-Proline	C_5_H_9_NO_2_	1.3329	*P* <0.01	0.5218	Down
2	L-Phenylalanine	C_9_H_11_NO_2_	1.4357	*P* <0.01	0.8922	Down
3	L-Isoleucine	C_6_H_13_NO_2_	1.4295	*P* <0.01	0.8742	Down
4	Piperidine	C_5_H_11_N	1.4627	*P* <0.01	0.7622	Down
**Linoleic acid metabolism**						
5	Gamma-Linolenic acid	C_18_H_30_O_2_	1.4264	*P* <0.05	1.2201	Up
6	9,10-DHOME	C_18_H_34_O_4_	1.6152	*P* <0.01	0.8576	Down
7	9-OxoODE	C_18_H_30_O_3_	1.7472	*P* <0.01	0.9578	Down
**Lysine degradation**						
8	S-Glutaryldihydrolipoamide	C_13_H_23_NO_4_S_2_	1.5437	*P* <0.01	0.5526	Down
9	(3S)-3,6-Diaminohexanoate	C_6_H_14_N_2_O_2_	1.4846	*P* <0.05	0.7140	Down
10	Glutaric acid	C_5_H_8_O_4_	1.1763	*P* <0.05	0.9446	Down
**Fatty acid degradation**						
11	Palmitic acid	C_16_H_32_O_2_	1.1059	*P* <0.05	0.9261	Down

a *HRFI, high residual feed intake; LRFI, low residual feed intake*.

b *VIP, variable importance in the projection*.

c *P-values are derived using a Student's t-test to assess the differences between HRFI and LRFI groups*.

d *Down, downregulated; up, upregulated*.

#### Correlation Among the Rumen Genera Bacteria, Significantly Differentially Abundant Rumen Metabolites, and the RFI Phenotype

**Figure 4:**
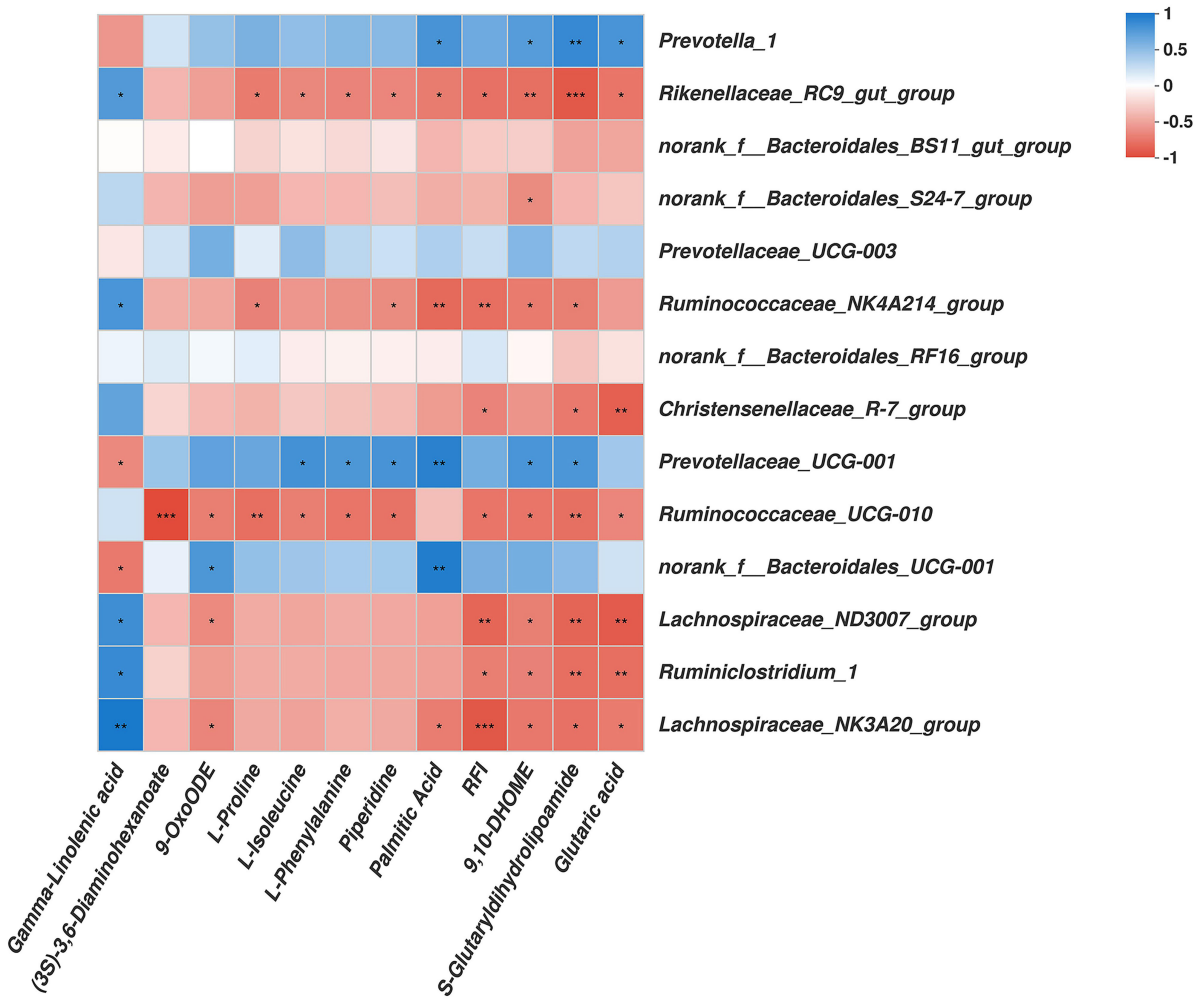
Correlation analysis among rumen genera bacteria, rumen metabolites affected by RFI, and the RFI. Cells are colored based on Spearman's correlation coefficient: Blue represents a positive correlation; red represents a negative correlation. Significant correlation: ***P ≤ 0.001, **0.001 < *P* ≤ 0.01; * 0.01 < *P* ≤ 0.05.

As shown in Figure 4, among the bacterial communities with a relatively high abundance and significantly differential bacteria at the genus level in the LRFI group, the genera *Rikenellaceae_RC9_gut_group, Ruminococcaceae_NK4A214_group, Christensenellaceae_R-7_group, Ruminococcaceae_UCG-010, Lachnospiraceae_ND3007_group, Ruminiclostridium_1*, and *Lachnospiraceae_NK3A20_group* were negatively associated with the RFI. Piperidine, 9,10-DHOME, S-Glutaryldihydrolipoamide, and Glutaric acid were positively associated with the genera *Prevotella_1*. The genus *Rikenellaceae_RC9_gut_group* was negatively associated with L-Proline, L-Isoleucine, L-Phenylalanine, Piperidine, Palmitic Acid, 9,10-DHOME, S-Glutaryldihydrolipoamide, and Glutaric acid. The genus *Ruminococcaceae_NK4A214_group* was negatively associated with L-Proline, Piperidine, Palmitic Acid, 9,10-DHOME, and S-Glutaryldihydrolipoamide. S-Glutaryldihydrolipoamide and Glutaric acid was negatively associated with the genera *Christensenellaceae_R-7_group*. The genus *Prevotellaceae_UCG-001* was positively associated with L-Isoleucine, L-Phenylalanine, Piperidine, Palmitic Acid, 9,10-DHOME, and S-Glutaryldihydrolipoamide. (3S)-3,6-Diaminohexanoate, 9-OxoODE, L-Proline, L-Isoleucine, L-Phenylalanine, Piperidine, 9,10-DHOME, S-Glutaryldihydrolipoamide, and Glutaric acid was negatively associated with the genus *Ruminococcaceae_UCG-010*. The genus *norank_f__Bacteroidales_UCG-001* was positively associated with 9-OxoODE and Palmitic Acid, but negatively associated with Gamma-Linolenic acid. Gamma-Linolenic acid was negatively associated with the genera *norank_f__Bacteroidales_UCG-001* and *Prevotellaceae_UCG-001*, while positively associated with the genera *Rikenellaceae_RC9_gut_group, Ruminococcaceae_NK4A214_group, Lachnospiraceae_ND3007_group, Ruminiclostridium_1*, and *Lachnospiraceae_NK3A20_group*. The genera *Lachnospiraceae_ND3007_group, Ruminiclostridium_1*, and *Lachnospiraceae_NK3A20_group* were all negatively associated with 9,10-DHOME, and S-Glutaryldihydrolipoamide. The genera *Lachnospiraceae_ND3007_group* and *Lachnospiraceae_NK3A20_group* were both negatively associated with 9-OxoODE, moreover, the genus *Lachnospiraceae_NK3A20_group* was negatively associated with Palmitic Acid.

## Discussion

### Animal Performance

As expected, the LRFI animals consumed less feed during the feedlot period, consistent with reports on steer performance (15). Improving feed efficiency is critical, because feed costs are the greatest driver of the profitability of beef production (37). Therefore, it is imperative that we select LRFI cattle that consume less feed without affecting their ADG, resulting in maximized profitability of the beef industry (1).

### Rumen Fermentative Parameters

The rumen microbiota mediates the energy available to the host ruminant through its fermentative activity, which suggests that it plays a role in feed efficiency (38). Shabat et al. (14) reported that efficient dairy cows had higher total VFAs than inefficient animals. Despite this, the present study did not detect a difference in rumen fermentative parameters between the HRFI heifers and the LRFI heifers, which agrees with the results of Welch et al. (15) in steers. Also, previous studies observed no relationship between rumen fermentation traits and the RFI phenotype of steers with different breeds. Interestingly, these results indicated that the dietary phase has a more pronounced influence on bacterial fermentation traits than the RFI phenotype (38, 39). Therefore, we speculated that the role of microorganisms would be more significant in the absorption of VFAs of the rumen epithelium in this study. Moreover, as described in the previous studies by Stewart et al. (40) and Bryant (41), ruminal VFA concentrations continued to decrease after fasting, especially a rapid decrease after feeding for 4 to 6 h, which explains our result that the rumen VFA concentration was lower than anticipated, because rumen liquid samples collected before morning feeding (15).

### Bacterial Diversity

Unlike other studies that found lower diversity and richness in the rumen bacteria of animals with high feed efficiency (14, 42), the present study did not observe differences in the ruminal microbial diversity of animals with high and low feed efficiency. The result of the present study corroborated the findings of earlier studies (16, 43, 44). These results indicated that a lower diversity microbiome may not necessarily equate to a more feed efficient ruminant and that microbial diversity is influenced by the chemical composition of the diet and breed, as previously reported (45).

### Significantly Differentially Abundant Rumen Bacteria

The observation of bacteria members related to fiber degradation in this study was not surprising given that the experimental diet had a concentrate:forage ratio of 5:5. In the present study, *Ruminococcaceae* was found in greater abundance in the rumen liquid of the most efficient heifers. Furthermore, the negative association of higher abundance of *Ruminococcaceae* with feed efficiency supports this result (12, 14). Members of the *Ruminococcaceae* members are well known to possess cellulolytic and hemicellulolytic activity, which produces acetate, butyrate, formate, and hydrogen (46). Therefore, the higher proportion of *Ruminococcaceae* likely resulted in increased energy utilization efficiency of animals with high feed efficiency. Additionally, a previous study reported that the population of *Rikenellaceae* in feces correlated negatively with feed efficiency (15), which partly supports our result of a higher population of *Rikenellaceae* members in the rumen liquid of the LRFI group. Lu et al. (47) reported that *Rikenellaceae* reduced fat by the synthesis of acetate and propionate. However, no differences were observed in butyrate and propionate concentrations in the rumen liquid between efficient and inefficient heifers in the present study. Therefore, whether *Rikenellaceae* improves feed efficiency by reducing fat needs to be verified by future studies. In addition, in the present study, the *Lachnospiraceae* population was also higher in the LRFI group, in agreement with previous observations (13, 14). Some members of *Lachnospiraceae* are major butyrate producers (48), and are thought to contribute to a healthy intestinal environment (49). Furthermore, the *Lachnospiraceae_ND3007* and *Lachnospiraceae_NK3A20* groups in this study were strongly associated with Linoleic acid metabolism and Lysine degradation, which prompted us to speculate that *Lachnospiraceae* improves the metabolic capability of Angus heifers in the LRFI group. In the present study, correlation analysis revealed that *norank_f__Bacteroidales_S24_7_group* was negatively associated with oxidative pro-inflammatory lipid metabolites of 9,10-DHOME (50). Similarly, Chen et al. (51) and Qi et al. (52) reported *norank_f__Bacteroidales_S24_7_group* as intestinal probiotics that had a negative correlation with intestinal inflammation. Our results further verified the anti-inflammatory function of the *Bacteroidales_S24-7_group*. Thus, a higher proportion of *norank_f__Bacteroidales_S24_7_group* might increase immunological function to promote feed efficiency in the LRFI group.

### Significantly Differentially Abundant Rumen Metabolites

The metabolome data revealed that the RFI alters the metabolite concentration of microorganisms in the rumen liquid, indicating that ruminal metabolism might be related to the ruminal microbiota. Our results showed that the RFI significantly altered the concentration of some important metabolites associated with protein digestion and absorption. In the rumen, amino acids are the degradation products of dietary or microbial proteins, and are the precursors of peptide and protein synthesis, which regulate certain metabolic pathways (53). Notably, L-proline, a functional amino acid, plays important roles in protein synthesis, structure, metabolism, and nutrition, as well as anti-oxidative reactions and immune responses (54). Downregulation of L-Proline might suggest more L-proline being used for microbial protein synthesis, which would be beneficial to animals in the LRFI group, as would the downregulation of L-Phenylalanine, L-Isoleucine, and Piperidine. Gamma-Linolenic acid, a kind of w-6 fatty acid, has anti-inflammatory effects (55). The upregulation of Gamma-Linolenic acid suggested that the LRFI group might have improved feed efficiency by improving their immune function. Furthermore, the LRFI group had lower levels of oxidative pro-inflammatory lipid metabolites, such as 9,10-DHOME and 9-OxoODE (50), providing further evidence of enhanced immune function. Lysine is the first or second limiting amino acid in beef cattle (56). The downregulation of Lysine degradation metabolites suggested that Lysine metabolism pathways were upregulated. We hypothesized that Lysine might be used to synthesize more microbial proteins, which would improve the feed efficiency of the LRFI group. Palmitic acid, a typical long-chain saturated fatty acid, has strong lipid toxicity and can cause excessive intracellular inflammatory reactions (57) and oxidative stress (58). Knight et al. (59) reported that microorganisms can synthesize palmitic acid using acetate. Thus, the downregulation of palmitic acid would not only reduce excess energy consumption, but also protect the health of cells, which in turn would improve the feed efficiency of the LRFI group.

### Correlations Among the Rumen Genera Bacteria, Significantly Differentially Abundant Rumen Metabolites, and the RFI Phenotype

Saleem et al. (60) reported that about 55–60% of the rumen fluid metabolites correlated with the rumen microbiota. We studied the correlation between the significantly differentially abundant ruminal metabolites and the genus level of the predominant rumen microbiota. Within the rumen microbiome, *Prevotella* has been recognized as the dominant bacterial genus (61), and has a documented role in the digestion of polysaccharides (62) and proteins (63). In agreement with our results, members of the family *Prevotellaceae* have been associated with inefficient dairy cows (13, 14). In the rumen, the *Prevotellaceae* family has the ability to degrade lignocellulose (64), and is involved in pectin and protein metabolism (65). In addition, *Prevotellaceae* plays an important role in degrading oligopeptides in ruminants (66). Xue et al. (67) reported that *Prevotella_1* affects the metabolism of amino acids in the rumen, because protein is a nitrogen source, which is essential for the growth of *Prevotella_1*. In our study, the proportion of the genus *Prevotella_1* was found to correlate strongly with Lysine degradation, and a higher proportion of *Prevotella_1* decreased the concentration of Lysine, which might have decreased the feed efficiency in the HRFI group. Liu et al. (68) found that *Prevotellaceae_UCG-001* produces anti-inflammatory metabolites, such as short-chain fatty acids (SCFAs). Knight et al. (59) reported that microorganisms can synthesize palmitic acid using acetate. However, our results showed that *Prevotellaceae_UCG-001* correlated positively with Palmitic acid, which can cause excessive intracellular inflammatory reactions (57). Therefore, the function of *Prevotellaceae_UCG-001* needs to be validated in future studies.

Tao et al. (69) reported that *Rikenellaceae_RC9_group* plays an important role in the degradation of structural carbohydrates. However, our results showed that *Rikenellaceae_RC9_group* correlated strongly with oxidative pro-inflammatory lipid metabolites of 9,10-DHOME (50) and the metabolism of Lysine, thus a higher proportion of *Rikenellaceae_RC9_group* might increase immunological function to promote the feed efficiency of the LRFI group. *Ruminiclostridium* spp. are best known for their cellulolytic activities. Loman et al. (70) reported that they might have a role in the gut-brain axis. Meanwhile, Zhao et al. (71) observed that *Ruminiclostridium* is important in controlling obesity development. Although their effects on host physiology have not been studied widely, our results suggest that they might have a role in Lysine metabolism in the rumen (70). Dai et al. (72) reported that *Christensenellaceae_R7_group* might be important in the degradation of hemicellulose and cellulose; however, in the present study, *Christensenellaceae_R7_group* was negatively associated with RFI, and might have a role in the metabolism of Lysine, which provides evidence supporting the higher feed efficiency in the LRFI group. Notably, Zhang et al. (73) reported that the proportion of *Ruminococcaceae_NK4A214_group* was related to the absorption of fat-soluble vitamins; however, our results suggested that it plays a role in the degradation of fatty acid, which might increase immunological function to promote the feed efficiency in the LRFI group. However, the mechanisms of *Lachnospiraceae_ND3007_group, Ruminiclostridium_1*, and *Lachnospiraceae_NK3A20_group* metabolism are not yet clear. In the present study, we found that these bacteria correlated negatively with the RFI phenotype, Linoleic acid metabolism, and Lysine degradation, but correlated positively with fatty acid degradation. It would be worth exploring the possibility that the proportion of these bacteria could affect the RFI phenotype.

## Conclusion

This study combined microbiome and metabolomic analyses to study the effects of high and low RFI Angus heifers on ruminal microbial communities and metabolites under the same feeding conditions. The genera *Rikenellaceae_RC9_gut_group, Ruminococcaceae_NK4A214_group, Christensenellaceae_R7_group, Ruminococcaceae_UCG-010, Lachnospiraceae_ND3007_group, Ruminiclostridium_1*, and *Lachnospiraceae_NK3A20_group* were negatively associated with RFI; both foundational and key species are associated with feed efficiency phenotype. Moreover, the RFI significantly altered the concentrations of ruminal metabolites involved in protein digestion and absorption, Linoleic acid metabolism, Lysine degradation, and Fatty acid degradation. In addition, our results also identified the relationship between rumen bacteria and metabolites. Integrative information about the interactions between the rumen microbial composition and metabolites in beef cattle with different RFI phenotypes could provide a better understanding of the ruminal microbial and metabolite functions, allowing the development of improved strategies to increase feed efficiency. Because of the individual variation of animals, in the future, samples from more cattle should be analyzed to confirm these findings. In addition, the mechanisms of the interactions among ruminal bacteria and rumen metabolism deserve further investigation.

## Data Availability Statement

The datasets presented in this study can be found in online repositories. The names of the repository/repositories and accession number(s) can be found at: https://www.ncbi.nlm.nih.gov/, PRJNA779020.

## Ethics Statement

The animal study was reviewed and approved by the Laboratory Animal Welfare and Animal Experiment Ethical Committee of China Agricultural University (Protocol No. AW08059102-2).

## Author Contributions

QM, ZZ, and HW designed the research. YL, WC, and CL conducted the research. YL analyzed the data and wrote the manuscript. ZZ had responsibility for the final content. All authors read and approved the final manuscript.

## Funding

This work was supported by grants from the National Natural Science Foundation of China (Grant Number: 31972593), the Government Purchase Service (Grant Number: 16200158), and the China Agricultural Research System (Grant Number: CARS-37).

## Conflict of Interest

The authors declare that the research was conducted in the absence of any commercial or financial relationships that could be construed as a potential conflict of interest.

## Publisher's Note

All claims expressed in this article are solely those of the authors and do not necessarily represent those of their affiliated organizations, or those of the publisher, the editors and the reviewers. Any product that may be evaluated in this article, or claim that may be made by its manufacturer, is not guaranteed or endorsed by the publisher.

## Abbreviations

RFI: residual feed intake
HRFI: high RFI
LRFI: low RFI
G/F: Gain-to-Feed Ratio
F/G: Feed-to-Gain Ratio
FCR: feed conversion rate
DMI: dry matter intake
ADG: average daily gain
VFAs: volatile fatty acids
SD: standard deviation
TVFAs: total VFAs
AP: ratio of acetate:propionate.
