# Supplementary material for: Rumen Microbiome and Metabolome of High and Low Residual Feed Intake Angus Heifers
Source: Front Vet Sci. 2022 Mar 25;9:812861. doi: 10.3389/fvets.2022.812861 (PMC8993041; doi:10.3389/fvets.2022.812861)
Supplement: Supplementary file 1 [file Data_Sheet_1.pdf]

Supplementary Material

1 Supplementary Figures and Tables

1.1 Supplementary Figures

Supplementary Figure 1 shows the ruminal bacteria composition of HRFI and LRFI Angus heifers. (A) the average relative abundances of phyla. (B) the average relative abundances of families. (C) the average relative abundances of genera. (relative abundance > 1% for all samples).

Supplementary Figure 2 shows the overlap of the total ion chromatogram of the QC sample in (A) the positive and (B) the negative ion modes, respectively. The results confirm the reliable repeatability and precision of the data obtained in the present study.

Supplementary Figure 3 shows samples examined by PCA following (A) the positive and (B) the negative mode ionization to provide a global overview of the differences among the metabolite data.

Supplementary Figure 1

A

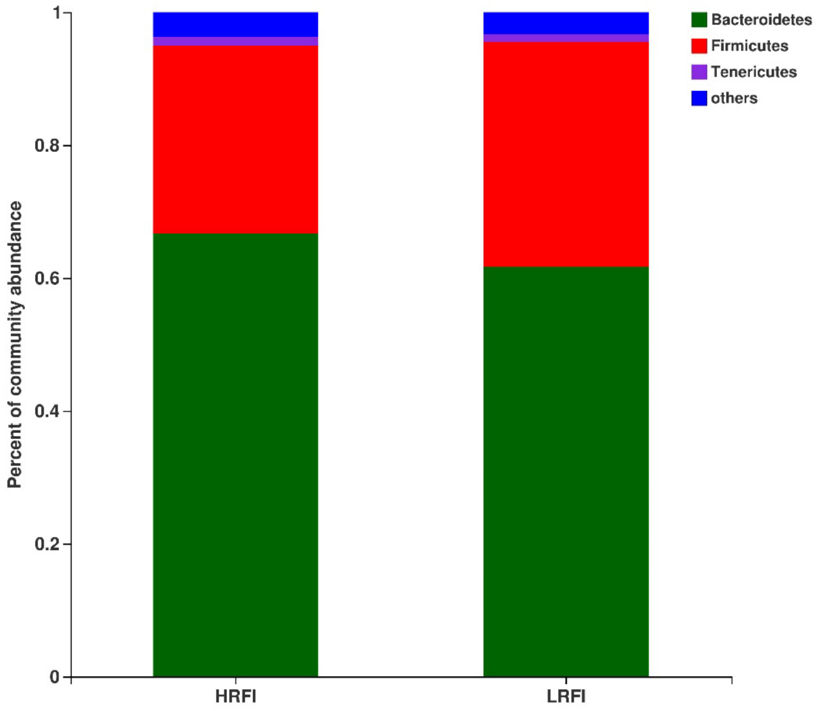

B

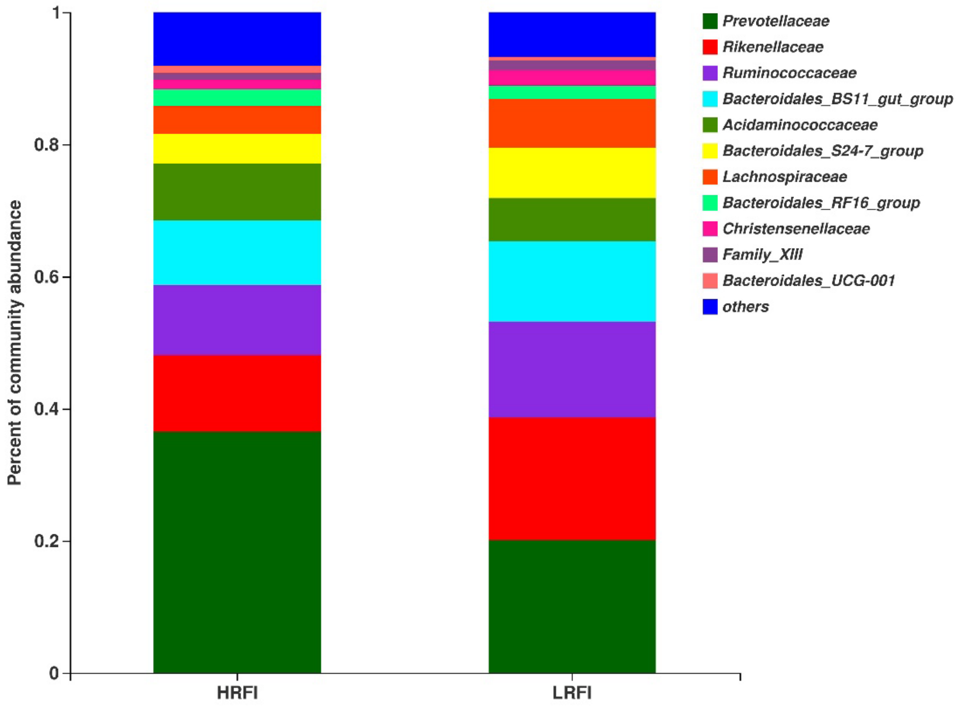
Percent of community abundance
0
0.2
0.4
0.6
0.8
1
HRFI
LRFI
Prevotellaceae
Rikenellaceae
Ruminococcaceae
Bacteroidales_BS11_gut_group
Acidaminococcaceae
Bacteroidales_S24-7_group
Lachnospiraceae
Bacteroidales_RF16_group
Christensenellaceae
Family_XIII
Bacteroidales_UCG-001
others

C

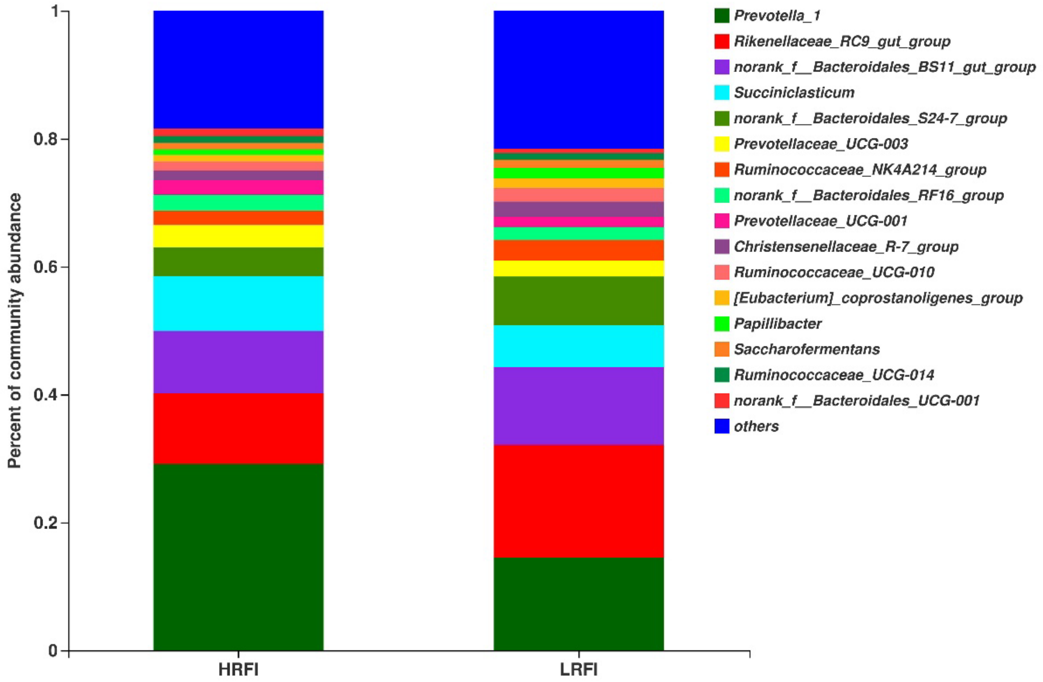
Percent of community abundance
0
0.2
0.4
0.6
0.8
1
HRFI
LRFI
Prevotella_1
Rikenellaceae_RC9_gut_group
norank_f__Bacteroidales_BS11_gut_group
Succiniclasticum
norank_f__Bacteroidales_S24-7_group
Prevotellaceae_UCG-003
Ruminococcaceae_NK4A214_group
norank_f__Bacteroidales_RF16_group
Prevotellaceae_UCG-001
Christensenellaceae_R-7_group
Ruminococcaceae_UCG-010
[Eubacterium]_coprostanoligenes_group
Papillibacter
Saccharofermentans
Ruminococcaceae_UCG-014
norank_f__Bacteroidales_UCG-001
others

Supplementary Figure 1. Stacked bar graphs of the average relative abundances of phyla (**A**), families (**B**), and genera (**C**) (relative abundance > 1% for all samples).

Supplementary Figure 2

A

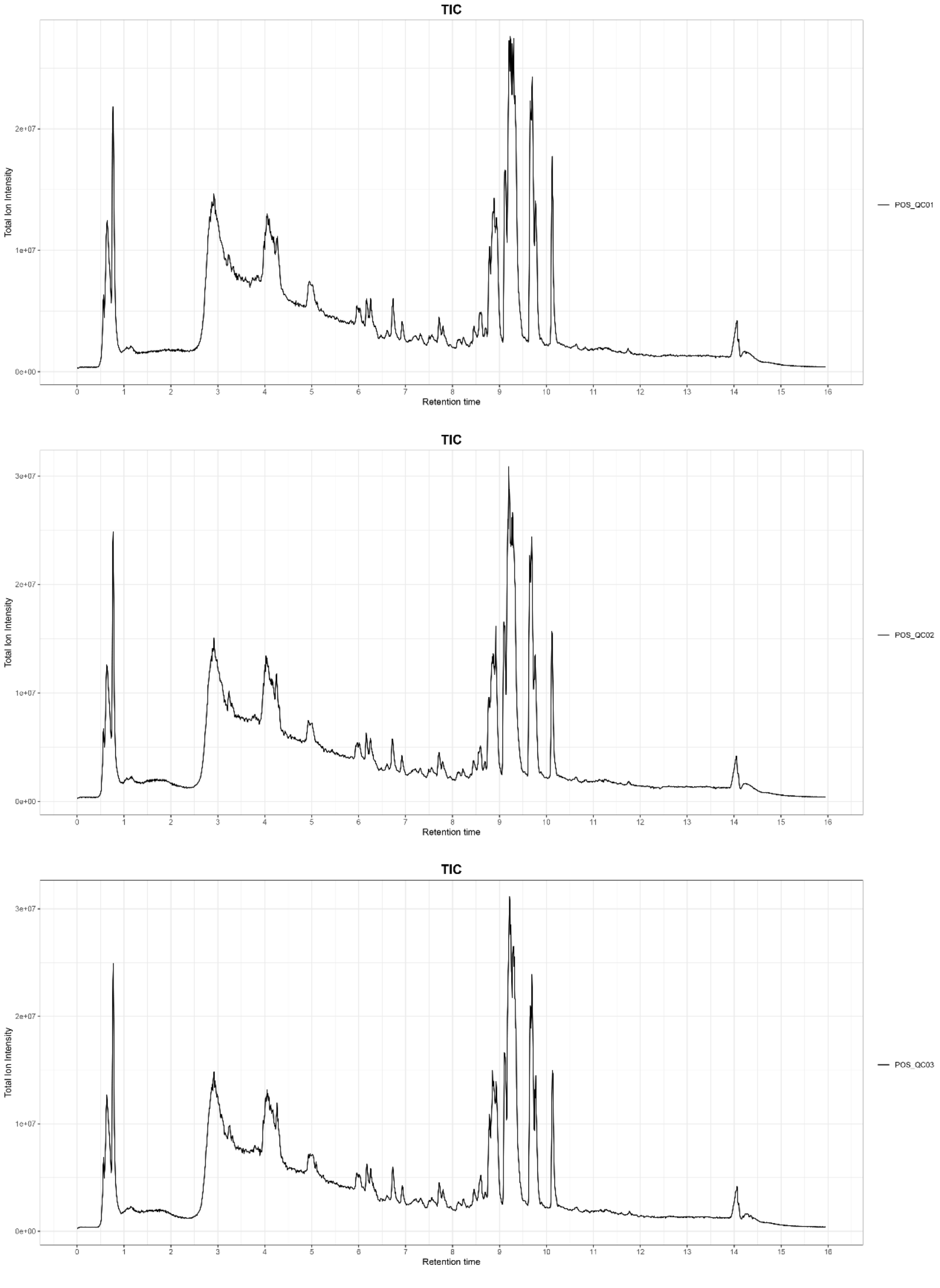

B

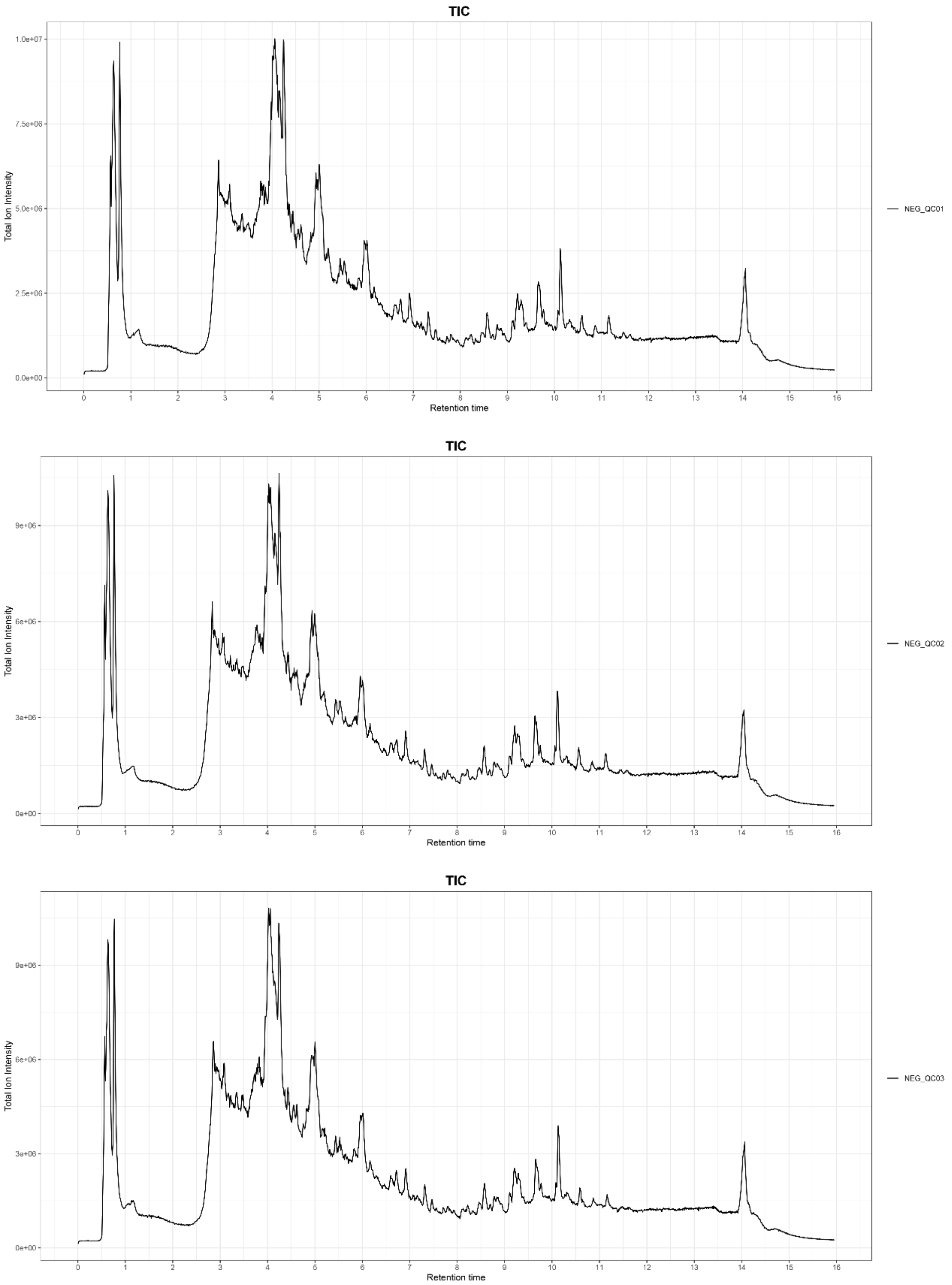

Supplementary Figure 2. LC-MS total ion chromatogram of the QC sample in **(A)** the positive ion mode and **(B)** the negative ion mode.

Supplementary Figure 3

A

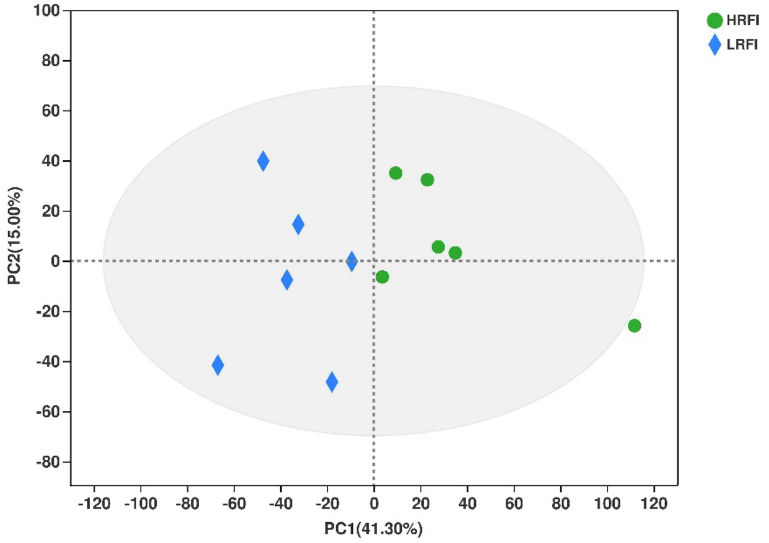

B

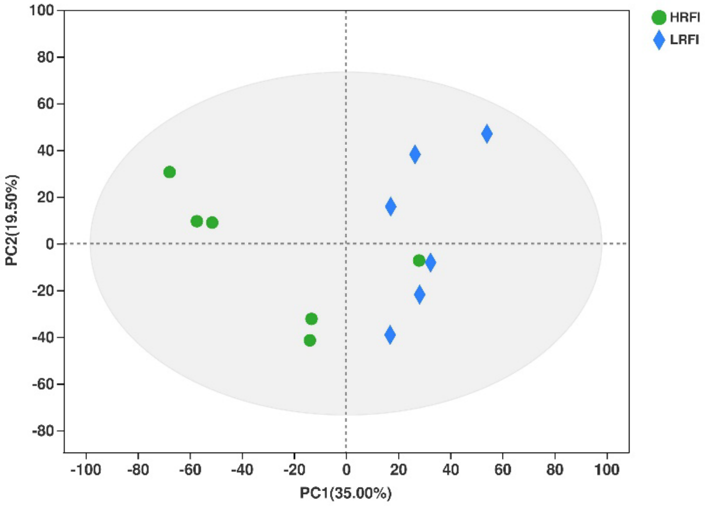

Supplementary Figure 3. The PCA plots of the ruminal samples corresponding to the HRFI and LRFI groups following **(A)** the positive and **(B)** the negative modes ionization.

1.2 Supplementary Tables

Supplementary Table 1. Ingredients and chemical composition of the experimental diet.

Item	Value
Ingredient composition, % DM	
Whole corn silage	30.00
Wheat shell powder	20.00
Corn	28.00
Cottonseed meal	10.50
Corn germ meal	10.00
Premix[1]	0.20
Salt	0.50
MgO	0.20
Limestone	0.60
Chemical composition (DM basis), %	
CP	13.25
NDF	43.62
ME, Mcal/kg	2.59

[1]Mineral supplement contains 0.198% cobalt as well as 0.9228% copper, 8.0376% iron, 0.0754% iodine, 5.8131% manganese, 0.0366% selenium, and 6.635% zine.

Supplementary Table 2. Residual feed intake (RFI) values of the 42 Angus heifers evaluated in the present study.

Animal number	RFI	Group [1]
**30**	**-1.951**	**LRFI**
**51**	**-1.823**	**LRFI**
**17**	**-1.662**	**LRFI**
**40**	**-0.998**	**LRFI**
**49**	**-0.825**	**LRFI**
**66**	**-0.703**	**LRFI**
57	-0.663	LRFI
3	-0.654	LRFI
62	-0.614	LRFI
1	-0.436	LRFI
86	-0.413	LRFI
15	-0.387	LRFI
10	-0.351	NS
11	-0.329	NS
56	-0.307	NS
44	-0.181	NS

72	-0.155	NS
5	-0.133	NS
71	-0.095	NS
80	-0.028	NS
8	0.008	NS
73	0.053	NS
12	0.217	NS
50	0.332	NS
14	0.350	NS
48	0.398	HRFI
24	0.411	HRFI
37	0.441	HRFI
13	0.461	HRFI
82	0.520	HRFI
55	0.541	HRFI
68	0.545	HRFI
2	0.614	HRFI
7	0.664	HRFI
55	0.709	HRFI
37	0.719	HRFI
**28**	**0.729**	**HRFI**
**29**	**0.757**	**HRFI**
**39**	**0.843**	**HRFI**
**45**	**0.904**	**HRFI**
**36**	**0.907**	**HRFI**
**64**	**1.584**	**HRFI**

[1] Bold lines represent the 12 heifers selected for evaluation in this study; NS: -0.38 < RFI < 0.38.

Supplementary Table 3. Animal performances of the 12 Angus heifers selected in the present study.

Animal number	Initial weight, kg	DMI[3], kg/d	ADG[4], kg/d	RFI, kg/d
**LRFI[1]**				
30	420.45	7.01	1.34	-1.951
51	424.31	7.15	1.20	-1.823
17	415.42	6.58	0.66	-1.662
40	386.15	7.74	0.88	-0.998
49	417.10	8.77	0.98	-0.825
66	412.21	8.72	1.00	-0.703
**HRFI[2]**				
28	431.41	8.82	1.09	0.729
29	434.62	8.80	1.00	0.757
39	429.50	10.12	0.86	0.843
45	399.68	10.38	0.93	0.904
36	433.32	9.05	0.74	0.907
64	425.15	9.91	0.66	1.584

[1] LRFI: low residual feed intake; [2] HRFI: high residual feed intake; [3] DMI: dry matter intake; [4] ADG: average daily gain.

Supplementary Table 4. Alpha diversity of ruminal microbial communities in composition of the HRFI and LRFI groups.

**Items** [1]	**HRFI**	**LRFI**	**SEM** [2]	***P*-value** [3]
Chao 1	1453.60	1480.70	71.42	0.53
Shannon	5.57	5.64	0.241	0.63
Simpson	0.013	0.012	0.007	0.79

[1] HRFI, high residual feed intake; LRFI, low residual feed intake. [2] SEM, standard error of the mean. [3] *P*-values are derived using a Student's t-test to assess the diferences between the HRFI and LRFI groups.
